# A deletion variant in *LMX1B* causing nail–patella syndrome in Japanese twins

**DOI:** 10.1038/s41439-024-00266-z

**Published:** 2024-02-29

**Authors:** Nozomu Kishio, Kazuhiro Iwama, Sayuri Nakanishi, Ryosuke Shindo, Masaki Yasui, Naoki Nicho, Atsushi Takahashi, Mana Kohara, Michisato Hirata, Takahiro Kemmotsu, Miki Tanoshima, Shuichi Ito

**Affiliations:** 1 Department of Maternal and Perinatal Center, Yokohama City University Medical Center, Yokohama, Japan; 2 Department of Pediatrics, Graduate School of Medicine, Yokohama City University, Yokohama, Japan; 3 Department of Clinical Genetics, Yokohama City University Medical Center, Yokohama, Japan

**Keywords:** Disease genetics, Genetic predisposition to disease

## Abstract

Nail–patella syndrome (NPS) is a hereditary disease caused by pathogenic variants in *LMX1B* and characterized by nail, limb, and renal symptoms. This study revealed a likely pathogenic *LMX1B* variant, NM_002316.4: c.723_726delinsC (p.Ser242del), in Japanese twins with clubfoot. The patients’ mother, who shared this variant, developed proteinuria after delivery. p.Ser242del is located in the homeodomain of the protein, in which variants that cause renal disease tend to cluster. Our findings highlight p.Ser242del as a likely pathogenic variant, expanding our knowledge of NPS.

Nail–patella syndrome (NPS; OMIM: 161200) is an autosomal dominant (AD) disease characterized by four major symptoms: nail dysplasia, patellar abnormalities, elbow dysplasia, and iliac horns. Other presentations of NPS patients include other skeletal abnormalities; nephropathy; hearing loss; and neurological, ophthalmological, and dental problems^1,2^. NPS exhibits full penetrance in heterozygous individuals, but its severity varies markedly. To date, more than 130 *LMX1B* pathogenic/likely pathogenic variants causative of NPS have been registered in ClinVar (https://www.ncbi.nlm.nih.gov/clinvar/, accessed 5 August, 2023). LMX1B is a member of the LIM homeodomain protein family; all proteins in this family have a homeodomain and a pair of LIM domains. The homeodomain binds to DNA to perform transcription factor functions by interacting with specific DNA elements in target genes^3^. A previous report showed an association between this homeodomain and nephropathy^2,3^. *LMX1B* has been reported to be essential for dorsoventral patterning during development, so the skeletal phenotype of NPS patients is considered to result from a deficiency in dorsoventral patterning regulated by *LMX1B*^3^.

The transcription factor LMX1B first gained prominence because of its involvement in dorsoventral axis formation during limb development^3^. One of the major determinants of the prognosis of NPS is kidney involvement, which is thought to be caused by arrested development of podocytes and glomeruli due to a pathogenic variant of *LMX1B*. NPS with kidney involvement has a broad spectrum of severity, ranging from asymptomatic proteinuria and/or hematuria to symptomatic kidney failure^4^.

In this paper, we describe a likely pathogenic variant of *LMX1B* identified by clinical genetic analysis of Japanese twins born to an NPS-affected mother. The pedigree of this family is shown in Fig. 1(i). III-3 was diagnosed with NPS in infancy and had typical symptoms, such as triangular deformities of the nail, underdeveloped patellae, hearing loss in the right ear, and asymptomatic hypercalciuria. Her proteinuria became apparent after delivery of the twins (described below). III-1 was diagnosed with NPS and presented with symptoms of malformed finger and toenails and elbow anomalies with difficulty extending. II-2 was also diagnosed with NPS, which presented as malformed nails.

**Fig. 1:**
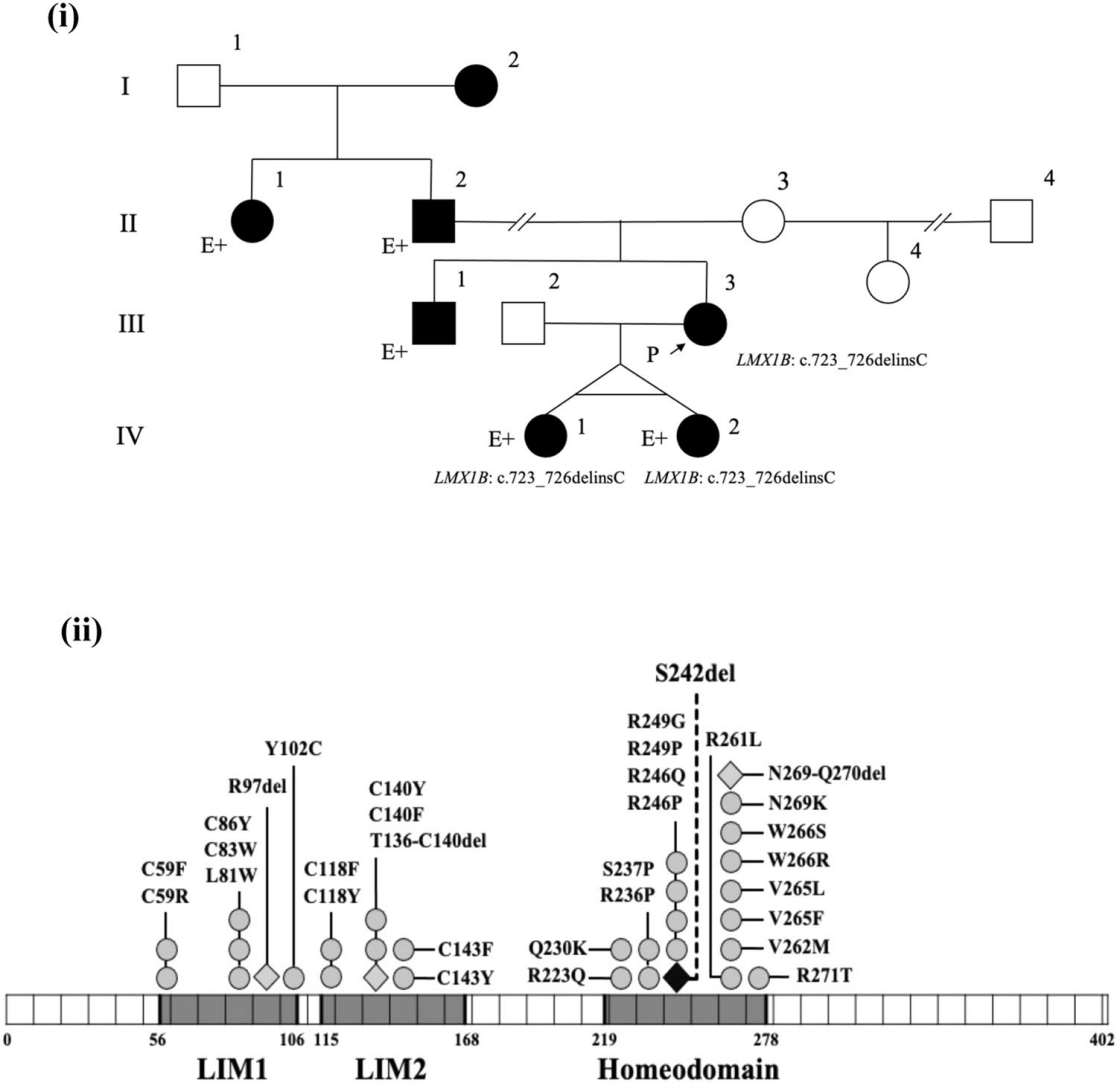
Pedigree diagram of the family and LMX1B pathogenic/likely pathogenic variants. (**i**) Pedigree diagram showing segregation of the *LMX1B* variant in an autosomal dominant manner. I-2, II-1, and II-2 had previously been diagnosed with nail–patella syndrome (NPS), although the clinical phenotypes were unclear. III-1 (older) had malformed finger and toenails and elbow anomalies with difficulty in extension. III-3 (younger, profoundly affected) was diagnosed with NPS in infancy and exhibited typical symptoms such as triangular appearance of the lunulae, underdeveloped patellae, loss of hearing in the right ear, and proteinuria developing after delivery. (**ii**) LMX1B pathogenic and likely pathogenic variants in NPS patients from ClinVar (https://www.ncbi.nlm.nih.gov/clinvar/). LMX1B is composed of 402 amino acids, described by line segments of 10 amino acids each. The gray squares indicate three domains in LMX1B: the LIM1 domain, LIM2 domain, and homeodomain (residues 56–106, 115–168, and 219–278, respectively). Some pathogenic (or likely pathogenic) missense variants (gray circles) and an in-frame deletion (gray diamond) were clustered within the homeodomain. p.Ser242del (black diamond and dashed line) is located in the homeodomain.

The affected mother (III-3) delivered monochorionic diamniotic (MD) monozygotic twins (IV-1 and IV-2) at 36 weeks and 4 days of gestation by cesarean section. Both IV-1 and IV-2 are female and weighed 2090 g and 1902 g at birth, respectively. The twins had ankle varus deformities (Supplementary Fig. A, B) with no other dysmorphic features. Approximately 18 h after birth, they were found to have asymptomatic hypoglycemia (blood glucose of ~30 mg/dL). The patients were admitted to the neonatal intensive care unit and intravenously infused with glucose via enteral feeding. After admission, their blood glucose levels were stable, and their oral intake gradually increased. Hence, continuous intravenous glucose supplementation was stopped for IV-1 and IV-2 on the 3^rd^ and 4^th^ days after birth, respectively. Subsequently, no recurrence of hypoglycemia occurred, and the twins were discharged on the 15^th^ day after birth. At the 1-month follow-up, no notable changes in their general condition were noted. However, the triangular deformities of the nails had become more pronounced (Supplementary Fig. C, D).

Although ankle varus deformities were not observed in other affected family members, the family history strongly suggested that the twins suffered from NPS. After obtaining informed consent, peripheral whole-blood samples were obtained from the twins for genetic analysis. Written informed consent was also obtained from the parents for the anonymized publication of the patients’ details.

The sequences of the protein-coding region of *LMX1B* along with the intronic boundary regions were analyzed by targeted next-generation sequencing using the hybrid capture method at Kazusa DNA Research Institute (Kisarazu, Chiba, Japan). The obtained nucleotide sequences were compared with published human genome reference sequences, and the presence or absence of low-frequency nucleotide substitutions and deletions/insertions of short nucleotide sequences was analyzed.

DNA sequencing revealed that the affected siblings (IV-1 and IV-2) harbored a heterozygous deletion-insertion [NM_002316.4: c.723_726delinsC (p.Ser242del)] in exon 4 of *LMX1B*, which is located on chromosome 9q33.3 (Fig. 1(ii)). c.723_726delinsC was not registered in either the Human Genome Mutation Database (HGMD) or ClinVar, but it was also absent from the control databases [the Genome Aggregation Database (gnomAD) and Tohoku Medical Megabank Organization (ToMMo)]. The identification of this variant was consistent with the analysis previously performed for their mother (III-1), and the combined annotation-dependent depletion score was 32^5^. In accordance with the American College of Medical Genetics and Genomics (ACMG) guidelines, c.723_726delinsC was classified as a likely pathogenic variant^5^.

In the present case, considering that the affected twins were MD monozygotic, we were concerned about similar development of kidney involvement. To follow up for their kidney-related issues, they were scheduled for regular outpatient consultation at our pediatric nephrology department in infancy. NPS with kidney involvement has various characteristic patterns, such as proteinuria, hematuria, and end-stage kidney failure^4^. In a previous study, kidney failure was found in 3% (3/123) of NPS patients^6^. This study also revealed the exacerbation of kidney involvement in the perinatal period^6^, with the mother developing proteinuria after delivery of the twins. Another study showed that NPS patients with variants in the homeodomain are more likely than those with LIM domain site variants to suffer from proteinuria^2^ and that variants in patients with severe nephropathy tend to cluster in specific regions of the homeodomain. In addition, pathogenic variants identified in LMX1B-related nephropathy patients were also clustered in the homeodomain^3^.

NPS patients require regular follow-up consultations at a nephrology department to track renal function in infancy or early childhood. It has been suggested that screening for nephropathy should be performed annually, including measurements of spot urine protein/creatinine or the albumin/creatinine ratio^4^. Since some NPS patients have an earlier onset of glaucoma than does the general population, consultation with an ophthalmologist to screen for signs and symptoms of glaucoma is recommended for these patients^6^.

Pathogenic variants in *LMX1B* result in skeletal dysplasia affecting dorsal tissues, especially the nail and patella. These variants are also significantly expressed in the dorsal mesenchyme of developing joints^7^, consistent with clubfoot. A report from the early 1990s on patients with clubfoot^8^ suggested that the presence of clubfoot in the affected twins in the present study was a symptom of NPS, although there was no family history of this condition.

The affected twins had the same symptoms, such as triangular deformities of the nails and clubfoot, that led to a diagnosis of NPS despite the abundant variation generally reported among NPS patients. In previous case reports of MD twins with AD diseases, namely, Ehlers–Danlos syndrome^9^ and maturity-onset diabetes of the young^10^, similar symptoms in the twins helped achieve a diagnosis via genetic analysis. In addition, a comparison between siblings and twins affected by autosomal dominant polycystic kidney disease showed that there was a significantly smaller difference in age at end-stage renal disease in twins than in siblings^11^. The presentation of similar clinical symptoms in MD twins, who share much of their genetic information, suggests that the phenotypic variation observed in individuals with the same genotype is influenced by other modifying factors.

In the patients reported here, we identified a likely pathogenic variant, c.723_726delinsC (p.Ser242del). Hamlington et al. reported 22 pathogenic variants, including p.Ser242del, in NPS patients^12^; however, they did not report the exact clinical manifestations of the NPS patients. Here, we have described the clinical manifestations of limb and kidney involvement, which are considered to be associated with p.Ser242del.

The affected mother was shown to have proteinuria as a result of the progression of kidney disease, with which p.Ser242del can be associated, considering its location within the variant hotspot in the *LMX1B* homeodomain. For the appropriate follow-up of NPS-affected children, consultations with nephrologists, orthopedists, and other specialists are needed. Continuous follow-up of patient growth, development, and symptoms, as well as clarification of the relationships between genetic variants and symptoms, are also needed in these cases. The expansion of a database containing this information is highly important for the future diagnosis and follow-up of genetic diseases.

## Supplementary information

Supplementary Figures

## Data Availability

The relevant data from this Data Report are hosted at the Human Genome Variation Database at 10.6084/m9.figshare.hgv.3368.
