# Supplementary material for: A deletion variant in LMX1B causing nail–patella syndrome in Japanese twins
Source: Hum Genome Var. 2024 Feb 29;11:10. doi: 10.1038/s41439-024-00266-z (PMC10904864; doi:10.1038/s41439-024-00266-z)
Supplement: Supplementary file 1 — Supplementary Figures [file 41439_2024_266_MOESM1_ESM.pptx]

## Slide 1
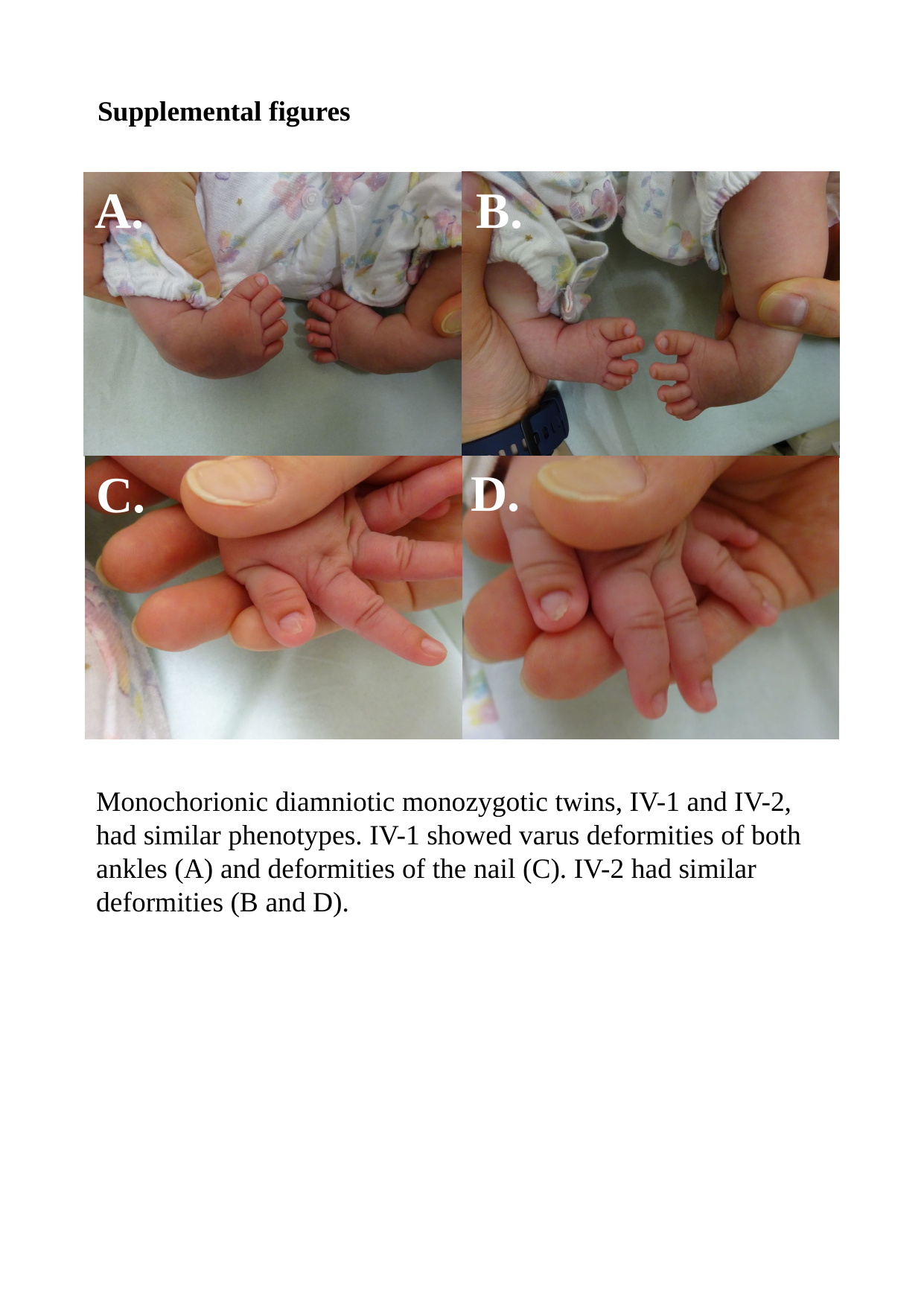

Supplemental figures
A.
B.
D.
C.
Monochorionic diamniotic monozygotic twins, IV-1 and IV-2, had similar phenotypes. IV-1 showed varus deformities of both ankles (A) and deformities of the nail (C). IV-2 had similar deformities (B and D).
